# Olfactory Sensitivity Is Related to Erectile Function in Adult Males

**DOI:** 10.3389/fcell.2020.00093

**Published:** 2020-02-27

**Authors:** Hui-yi Deng, Jia-rong Feng, Wen-hao Zhou, Wei-feng Kong, Gong-chao Ma, Teng-fei Hu, Shao-ge Luo, Yu Xi, Yan Zhang, Qin-tai Yang

**Affiliations:** ^1^Department of Otorhinolaryngology-Head and Neck Surgery, The Third Affiliated Hospital, Sun Yat-sen University, Guangzhou, China; ^2^Department of Infertility and Sexual Medicine, The Third Affiliated Hospital, Sun Yat-sen University, Guangzhou, China; ^3^Department of Urology, The First Affiliated Hospital of Guangzhou Medical University, Guangzhou, China; ^4^Department of Allergy, The Third Affiliated Hospital, Sun Yat-sen University, Guangzhou, China

**Keywords:** rhinologic diseases, erectile dysfunction, olfactory sensitivity, vomeronasal organ, Sniffin’ Sticks test, IIEF-5

## Abstract

**Background:**

The olfactory system influences human social behavior, in particular the selection of a spouse. However, there is currently a lack of clinical research on the relationship between the olfactory system and erectile dysfunction (ED) in adult males.

**Aim:**

We explored the association between olfactory sensitivity and erectile function and its possible mechanisms.

**Results:**

A total of 574 patients, adult males aged between 19 and 42 years, diagnosed with ED in the Department of Infertility and Sexual Medicine of the Third Affiliated Hospital of Sun Yat-sen University from 2015 to 2018 were analyzed retrospectively. Among them, 115 patients (20.03%) had rhinologic diseases (RDs). In addition, in 201 adult male patients who underwent nasal surgery in the ENT department from 2012 to 2016, including 29 (14.43%) with ED, nasal congestion, nasal discharge, and hyposmia were the most common complaints based on the numerical rating scale (NRS). Furthermore, a prospective study was performed in a total of 102 sequential outpatients (male adults) with RD only (*n* = 46), ED only (*n* = 42) and both RD and ED (*n* = 14) in 2019, together with 40 healthy (male adults) volunteers as controls. The results showed that ED patients with RD had severe nasal discomfort and decreased erectile function (*P* < 0.0001). The olfactory sensitivity of patients with ED was lower than that of the controls, and patients with both ED and RD had the worst olfactory sensitivity (*P* < 0.0001). Spearman correlation analyses showed that sense of smell was positively correlated with the International Index of Erectile Function-5 score (*R* = 0.507, *P* ≤ 0.0001) and the Erection Hardness Scale score (*R* = 0.341, *P* < 0.0001). Logistic regression analyses showed that having an olfactory disorder (OD), RD, age, and visual analog scale (VAS, over 5) score were risk factors for ED outcome, indicating that OD patients had a 16.479-fold increased risk for an ED outcome (*P* < 0.05).

**Conclusion:**

A significant correlation was detected between olfactory sensitivity and erectile function in adult males. In particularly, impairment of olfactory sensitivity is more common in patients with both ED and RD than in patients suffering from a single disease.

## Introduction

The olfactory system has a number of features associated with human ingestion, including the modulation of appetite, detection and identification of foods suitable for eating, and rejection of inedible foods (Stevenson, 2010). Olfaction plays a central role in mammalian sexual behavior as well (Pfeiffer and Johnston, 1994; Capparuccini et al., 2010). Olfactory regulation of social communication is vital to reproductive behavior, as it mediates emotional detection, inbreeding avoidance, and the selection of a spouse (Stevenson, 2010).

Previous studies have demonstrated that patients with rhinologic diseases (RDs), such as nasal polyps (NPs), rhinosinusitis (CRS), and allergic rhinitis (AR), have decreased erectile function, which improves significantly after treatment. Some clinical studies have shown that men with high olfactory sensitivity have high sexual desire and increased sexual experience. Therefore, olfaction appears to contribute to sexual arousal and sexual behavior, and people with impaired olfaction cannot benefit from olfaction increasing their sexual experience. In fact, the quality of life of patients with olfactory disorders (ODs) declines, affecting certain aspects of daily life, including social interaction and sexual contact (Croy et al., 2014). In a broad Internet-based survey of chemosensory impairment, more than 50% of participants complained that their chemosensory disorders had negative effects on their sexual activity (Merkonidis et al., 2015). One study showed that sexual desire decreased after olfactory loss in a large number of patients, moderated by the severity of olfactory symptoms and by depression. This effect is more pronounced in males than in females (Gudziol et al., 2009). ODs are often accompanied by a loss of mood and an impaired sexual life and social relationships, which makes patients more depressed and more dependent on their partners than controls (Schafer et al., 2019). In addition, better olfaction is related to better sexual experience. In healthy adults, the more sensitive the sense the smell, the greater the sexual pleasure and the more frequent the orgasm (Bendas et al., 2018). Another study showed a higher incidence of erectile dysfunction (ED) in patients suffering from NP (Gunhan et al., 2011). Surgery can improve the sexual functioning and sleep quality of patients with CRS and NPs, which indicates that CRS and NPs are independent risk factors for ED (Tai et al., 2016). Finally, patients with AR have a higher risk for ED in the future (Su et al., 2013).

These studies suggest a relationship between ODs and sexual behavior that may be regulated by social insecurity, depression, or other comorbidities. Patients with a combination of RDs have impaired erectile function, which improves when the RDs are treated or cured. To date, no study has investigated the direct effects of different manifestations of olfactory function on erectile function in males. In this study, we investigated the relationship between olfactory function and erectile performance in a male population. We speculated that higher olfactory sensitivity would be associated with better erectile function. We used the Erection Hardness Scale (EHS), the International Index of Erectile Function-5 (IIEF-5), a visual analog scale (VAS), and the Sniffin’ Sticks test (Hummel et al., 1997) to fully assess nasal discomfort and olfactory sensitivity.

## Materials and Methods

### Participants

Our study was carried out in accordance with the Declaration of Helsinki. Ethics approval was obtained from the institutional review boards of the Third Affiliated Hospital, Sun Yat-sen University, Guangzhou, China. All participants signed informed consent.

This study consisted of a retrospective cohort study with symptoms analysis and a prospective study with clinical measurements of olfactory sensitization and erectile functions in male adult patients. The demographic information of retrospective cohort study patients is shown in Table 1, for those of prospective study are described in Table 2.

**TABLE 1:** The demographic information and symptoms analysis of the retrospective cohort study.

**Characteristics**	**ED**	**RD**
Original sample size	613	234
Qualified for inclusion	574	201
Age, mean (range)	30 (18–48)	31 (22–51)
Complication (*n*, %)	With RD(115, 20.03)	With ED (29, 14.43)
**NRS for RD patients, mean (range)**		
Nasal congestion	7 (4–9)	8 (5–10)
Nasal discharge	7 (3–10)	7 (6–10)
Hyposmia	5 (5–9)	6 (4–10)

*NRS, numerical rating scale; RD, rhinologic disease; ED, erectile dysfunction.*

**TABLE 2:** Clinical characteristics, measures of olfactory sensitivity and erectile function of the four group patients in the prospective study.

**Characteristics**	**Controls**	**RD only**	**ED only**	**RD with ED**
Sample size	40	46	42	14
Mean age, (range)	26 (19–36)	27 (18–52)	30 (21–38)	30 (20–44)
IIEF-5	22.60 ± 1.23	21.28 ± 8.48	11.96 ± 5.61*	9.86 ± 6.67*^#^
EHS	3.70 ± 0.47	3.76 ± 0.43	2.62 ± 0.73*	2.25 ± 0.89*^#^
VAS scale	1.95 ± 1.50	5.61 ± 1.39	3.19 ± 1.87*	6.25 ± 1.88*^#^
**Sniffin’ Sticks test**				
Discrimination test	11.30 ± 1.63	11.61 ± 1.42	11.05 ± 1.66	9.50 ± 3.68*^#[*dollar*]^
Identification test	11.95 ± 1.90	11.50 ± 2.47	10.55 ± 1.29*	9.21 ± 3.56*^#[*dollar*]^
TDI score	35.80 ± 3.11	32.91 ± 5.39	31.46 ± 2.64*	25.56 ± 9.59*^#[*dollar*]^

RD, rhinologic disease; ED, erectile dysfunction; IIEF-5, International Index of Erectile Function-5; EHS, Erection Hardness Scale; VAS, visual analog scale; data are given as mean ± SD or *n* (percentage). *P-*values are determined by ANOVA test unless mentioned otherwise. “*” means the *P*-value is less than 0.05 and compared with the control group. “^#^” means compared with the ED only. “^$^” means compared with the RD only.

In the first part of study, the patients with ED who were admitted to the Department of Infertility and Sexual Medicine of the Third Affiliated Hospital of Sun Yat-sen University from 2015 to 2018 were included. We collected their data of nocturnal penile tumescence and rigidity (NPTR), records of outpatient visits, and prescription data for all patients with ED. Among the 613 ED patients, following patients were excluded due to organic ED (*n* = 19) based on NPTR, celibate patients (*n* = 6), mental disorder (*n* = 8) sever systemic disorders (*n* = 4) and patients older than 55 years (*n* = 2). The remaining 574 patients met the following inclusion criteria of physical ED based on NPTR, no other systemic disorders, aged between 18 and 55 years, ability to communicate, and detailed information at follow-up. The 115 patients who were diagnosed with RD in the outpatient clinic were selected and asked about their nasal symptoms using numerical rating scale (NRS), a 11-point rating scale (0–10) by phone, including nasal congestion, nasal discharge, sneezing, postnasal drip, loss of sense of smell, facial pain/stress, and headache.

In addition, we reviewed outpatient data from 234 adult male patients with RD who underwent nasal surgery in our hospital from January 2014 to December 2016. According to the inclusion criteria (without other severe systemic disorders, aged between 18 and 55 years, able to communicate, detailed information to follow-up, and no concurrent upper respiratory infection), 35 patients were diagnosed with ED, 6 patients had organic ED based on NPTR were excluded. Finally 201 patients proceeded for a telephone follow-up. Among them 29 patients with RD (non-organic ED) were asked about their nasal symptoms and severity which were graded by NRS.

In the prospective study (the second part), a total of 188 sequential adult male outpatients who visited the Departments of Infertility and Sexual Medicine and Otorhinolaryngology at the Third Affiliated Hospital of Sun Yat-sen University in 2019 were recruited. After screening with the same inclusion and exclusion criteria (in the first part), 102 patients were finally proceeded for all measurements including IIEF-5, EHS, the nasal symptoms VAS, and the Sniffin’ Sticks test. Then, they were further divided into three groups: RD only, ED only, and RD with ED. Meanwhile, 40 healthy volunteers with age- and gender-matched graduate students or medical staff were recruited as a control group.

### Nocturnal Penile Tumescence and Rigidity

As we reported previously (Zou et al., 2019), all patients underwent measurement of NPTR over two consecutive nights in the sleep unit of our clinic. Patients were prohibited from engaging in any activity that could interfere with sleep, including smoking; drinking tea, coffee, or alcohol; or taking hypnotics. A RigiScan Plus device (GOTOP Medical, Saint Paul, MN, United States) was strapped to the patient’s thigh. Two self-calibrating loops were attached to the penis, with one loop at the tip and the other at the base. Data collected included the number of effective erectile events, total erection time, tumescence-activated units, rigidity-activated units, average event rigidity, and duration of erectile episodes with rigidity ≥60%. In accordance with EAU guidelines regarding male sexual dysfunction (Hatzimouratidis et al., 2010), an effective erectile event was defined as an erectile episode with penile tip rigidity ≥60% and a duration of no less than 10 min. Moreover, a patient was considered to have normal erectile function when at least one effective erectile event was recorded over two consecutive nights of measurements, indicating non-organic ED. If a patient had mechanical problems or a sleep disorder, or if monitoring time was <6 h, the patient was retested and excluded from the study.

### Erectile Dysfunction

#### Erection Hardness Scale Score

Participants were asked to fill out the EHS for a subjective measurement of erection hardness. This questionnaire was originally used to assess the efficacy of sildenafil citrate for recovering erectile function (Goldstein et al., 1998). Then it was validated and standardized by Mulhall et al. (2007) as a reliable instrument for assessing erection hardness in clinical trials.

#### International Index of Erectile Function-5

Participants were asked to complete the IIEF, a brief, simple tool used to evaluate certain aspects of sexual function in adult males (Rosen et al., 1997). It contains five items on erection confidence and firmness, maintenance ability and frequency, and satisfaction. Each item is rated on a 5-point ordinal scale, where lower scores represent poorer erectile performance. The scores of all items are summed for an overall score ranging from 5 to 25, where a lower score represents more severe ED. The IIEF is used to diagnose and classify ED based on scores obtained on the erectile function domain: absence of ED (score: 22–25), mild (score: 17–21), mild to moderate (score: 12–16), moderate (score: 8–11), and severe (score: 5–7). These categories are based on our clinical understanding of the scores (Rosen et al., 1999).

#### Numerical Rating Scale for Nasal Disorders

In the retrospective cohort study, all participants underwent a phone call session to answer trial questions (“What is your pain level for your nasal symptoms?”) using the 11-point NRS, where 0 is not painful and 10 is the most worse painful (Kazi et al., 2019).

#### Visual Analog Scale for Nasal Symptoms

In the prospective study, RD was categorized as mild, moderate, or severe based on the total VAS score, with anchor points of 0 (not troublesome) and 10 (worst possible trouble): mild = 0–3, moderate ≥3–7, and severe ≥7–10 (Fokkens et al., 2012). To evaluate severity, we asked the patient to indicate on the VAS his answer to the question “How troublesome are your nasal disease symptoms?”

#### Sniffin’ Sticks Test

We used the Sniffin’ Sticks test (Burghart, Wedel, Germany) to evaluate the olfactory sensitivity of the participants (Hummel et al., 1997). The sets were performed in a particular order using odorant (phenylethyl alcohol) assembled pens. The test is a multiple forced-choice test that is performed in a ventilated and quiet room. The open pen was positioned 2 cm in front of the nose for one to two breaths. Each pen was used once with an interval of at least 30 s to prevent olfactory desensitization. Participants were blindfolded to reduce the possibility of visual detection. Three pens were positioned randomly for 3 s. Two pens contained an odorless solvent and the third contained a diluted odorant that was to be identified by the participant. We determined odor thresholds using the single staircase method (Bendas et al., 2018), starting at the lowest dilution and moving in the direction of increasing odor intensity. If participants correctly identified the odorant-containing pen two successive times, a reversal of the staircase was triggered; the opposite happened when the target pen was not identified properly. The threshold estimate was the mean of the last four staircase reversal points of the seven reversals. Higher values indicated higher olfactory sensitivity.

### Statistical Analyses

Analyses of variance were performed to compare mean values for clinical characteristics (age), nasal and sexual outcome variables (VAS, IIEF-5, and EHS scores), and olfactory sensitivity (Sniffin’ Sticks test: threshold test, discrimination test, identification test, and TDI score) among the four groups (ED, RD, ED with RD, and healthy controls; Table 2).

Spearman correlation analyses were used to assess potential relationships between the odor threshold (Sniffin’ Sticks test) and measures of erectile function (IIEF-5 and EHS) and nasal symptoms (VAS score) in each group of patients (Table 3).

**TABLE 3:** Spearman correlational analysis between olfactory sensitivity and VAS score, IIEF-5, EHS.

**Measurements of sexual function and nasal symptoms**	**Olfactory sensitivity via Sniffin’ Sticks test**			
	**Threshold test**	**Discrimination test**	**Identification test**	**TDI score**
**IIEF-5**				
Control	*R* = −0.153	*R* = 0.133	*R* = 0.449	*R* = 0.184
	*P* = 0.347	*P* = 0.413	*P* = 0.004	*P* = 0.255
RD	*R* = 0.328	*R* = 0.117	*R* = 0.329	*R* = 0.425
	*P* = 0.026	*P* = 0.438	*P* = 0.025	*P* = 0.003
ED	*R* = 0.102	*R* = 0.111	*R* = 0.076	*R* = 0.120
	*P* = 0.355	*P* = 0.316	*P* = 0.491	*P* = 0.277
RD + ED	*R* = 0.224	*R* = 0.072	*R* = 0.176	*R* = 0.052
	*P* = 0.251	*P* = 0.718	*P* = 0.371	*P* = 0.794
Combined	*R* = 0.421	*R* = 0.195	*R* = 0.409	*R* = 0.507
	*P* = 0.000	*P* = 0.006	*P* = 0.000	*P* = 0.000
**EHS**				
Control	*R* = −0.182	*R* = −0.164	*R* = 0.423	*R* = 0.143
	*P* = 0.261	*P* = 0.313	*P* = 0.007	*P* = 0.378
RD	*R* = 0.293	*R* = 0.137	*R* = 0.198	*R* = 0.254
	*P* = 0.048	*P* = 0.363	*P* = 0.187	*P* = 0.089
ED	*R* = 0.079	*R* = 0.045	*R* = −0.087	*R* = −0.063
	*P* = 0.474	*P* = 0.681	*P* = 0.429	*P* = 0.571
RD + ED	*R* = 0.196	*R* = −0.070	*R* = 0.134	*R* = 0.055
	*P* = 0.317	*P* = 0.723	*P* = 0.497	*P* = 0.782
Combined	*R* = 0.293	*R* = 0.125	*R* = 0.320	*R* = 0.341
	*P* = 0.000	*P* = 0.098	*P* = 0.000	*P* = 0.000
**VAS socre**				
Control	*R* = −0.017	*R* = 0.430	*R* = −0.080	*R* = 0.197
	*P* = 0.916	*P* = 0.006	*P* = 0.625	*P* = 0.223
RD	*R* = 0.025	*R* = −0.084	*R* = −0.232	*R* = −0.159
	*P* = 0.867	*P* = 0.752	*P* = 0.121	*P* = 0.292
ED	*R* = 0.154	*R* = −0.006	*R* = −0.020	*R* = 0.097
	*P* = 0.161	*P* = 0.959	*P* = 0.854	*P* = 0.378
RD + ED	*R* = −0.007	*R* = 0.142	*R* = 0.143	*R* = 0.096
	*P* = 0.97	*P* = 0.471	*P* = 0.468	*P* = 0.626
Combined	*R* = −0.173	*R* = 0.022	*R* = −0.027	*R* = −0.096
	*P* = 0.021	*P* = 0.769	*P* = 0.716	*P* = 0.203

*RD, rhinologic disease; ED, erectile dysfunction; IIEF-5, International Index of Erectile Function-5; EHS, Erection Hardness Scale; VAS, visual analog scale.*

Logistic regression was used to specify which moderator variables predicted change in erectile function. All moderator variables (age, VAS score, and ED or RD) were included as predictors (Table 4).

**TABLE 4:** The logistic regression analysis for erectile dysfunction.

**Risk factors**	**OR**	**95% CI**	***P-*value**
Age	1.099	(1.037, 1.164)	0.001
VAS	1.468	(1.176, 1.833)	0.001
**With OD**			
NO	–	–	–
Yes	17.479	(5.713, 53.477)	0.000
**With RD**			
NO	–	–	–
Yes	0.031	(0.009, 0.109)	0.000

*OD, olfactory disorder.*

*P* < 0.05 was considered significant. IBM SPSS^®^ version 21 (IBM, Armonk, NY, United States) was used for all analyses.

## Results

### ED With RD Complications

In the retrospective analyses of 574 patients, 115 patients (20.03%) had RDs, including 39 patients (33.91%) of AR, 23 patients (20%) of deviated septum, 18 patients (15.65%) of CRS, 9 patients (7.83%) of NPs, and 23 patients (22.61%) of chronic rhinitis. There were 341 effective responses during telephone follow-ups, and the main complaints were nasal congestion, nasal discharge, and hyposmia on the VAS score (>5), accounting for 82.42, 61.54, and 10.99% of the complaints, respectively.

### Rhinologic Disease With ED Complications

A total of 201 patients undergoing nasal surgery, 29 patients (14.43%) were diagnosed with ED. All of them were contacted during the telephone follow-up. Nasal congestion, nasal discharge, and a decrease in olfactory sensitivity were the most common complaints, accounting for 85.19, 62.07, and 22.22% of the complaints, respectively.

### The Relationship Between Erectile Function and Olfactory Sensitivity in Adult Males

One hundred and two patients and 40 healthy controls who were admitted in 2019 completed the IIEF, EHS, VAS, and the Sniffin’ Sticks test to elucidate the relationship between erectile function and olfaction in males. ED patients with RD had increased nasal discomfort and worse erectile function than patients with ED (*P* < 0.0001; Table 2). The olfactory sensitivity of patients with ED (threshold test: 9.86 ± 1.77, discrimination test: 11.05 ± 1.66, identification test: 10.55 ± 1.29, and TDI score: 31.46 ± 2.64) was lower than that of the healthy controls (threshold test: 12.55 ± 1.96, discrimination test: 11.30 ± 1.63, identification test: 11.95 ± 1.90, and TDI score: 35.80 ± 3.11), and patients with both ED and RD had the worst olfactory sensitivity (threshold test: 9.86 ± 1.77, discrimination test: 9.50 ± 3.68, identification test: 9.21 ± 3.56, and TDI score: 25.56 ± 9.59; *P* < 0.001; Table 2). A positive correlation was detected between olfactory sensitivity and IIEF-5 score (*R* = 0.507, *P* < 0.001) and EHS score (*R* = 0.341, *P* < 0.0001; Table 3).

### Factors Influencing Hyposmia

Logistic regression analyses were performed to identify risk factors for ED (Table 4). Patients with olfactory disease or RD, those who were older, and those with a higher VAS score were at higher risk for ED. Olfactory disease was associated with a 16.479-fold increase in ED outcomes (*P* < 0.0001). Older patients and people suffering from nasal discomfort had a 0.099 times and a 0.468 times increased risk for ED, individually (*P* < 0.01). We plotted the relationship between RD, ED, and olfactory disease and found that patients with RD with ED had a higher incidence of olfactory disease. This suggests that nasal congestion, runny nose, and erectile function caused by RD are positively correlated with olfactory disease. Combinations of ED and RD resulted in additive effects.

## Discussion

The predictive correlation between olfaction and sexual desire and sexual experience has been confirmed (Bendas et al., 2018; Schafer et al., 2019). Our retrospective analyses found that patients with ED were more likely than healthy controls to suffer from RD at the same time. A decline in olfactory sensitivity is often overlooked, because a minor decline in smell does not have a significant impact on daily life. Yet a recent study indicated that patients who complain of OD experience a significantly reduced quality of life regarding paid employment, household work, and social and family life (Bramerson et al., 2007). The present study found that nearly 15% of patients with RD have ED and that patients with RD and ED have the worst olfactory sensitivity. In summary, RDs are closely related to ED, which is consistent with previous results (Su et al., 2013). Olfactory sensitivity is associated with RD and ED. This is consistent with a study reporting that men who are born without a sense of smell exhibit a markedly reduced number of sexual relationships. The olfactory function test introduced in this study showed that olfactory sensitivity decreased in patients with RD and ED and that the olfactory threshold was significantly higher than in healthy controls (Table 2). Moreover, sense of smell decreased significantly, nasal discomfort increased, and overall erectile function was unsatisfactory in patients who suffered from both RD and ED. These results are consistent with previous findings of patients diagnosed with dysosmia losing sexual desire (Croy et al., 2013; Gudziol et al., 2009).

We detected a significant correlation between olfactory threshold and ED. Unfortunately, we did not observe a relationship between olfactory function and the severity of nasal discomfort. Studies have shown that the sense of smell is closely associated with the sexual process. Patients with decreased olfactory sensitivity are more likely to have ED. Therefore, smell could be considered an initiator of sexual activity, and penile erection is the effector. According to the literature, the normal operation of the effector results from a functional vomeronasal organ (VNO), because the human VNO provides important information on inhibiting chemical sensation, promoting heterosexual contact, and preventing inappropriate mating. Foltan and Sedy (2009) hypothesized that the adult VNO is indispensable in preventing the selection of an inappropriate spouse. Human pheromones extracted from armpit sweat and tears control ovulation, temper, hormone secretion, and attraction to different genetic partners (Wysocki and Preti, 2004). Periovulatory odors increase testosterone and cortisol levels in adult males, producing strong sexual desire (Cerda-Molina et al., 2013). Croy reported that the hypothalamus becomes activated in men with medium androstenedione concentrations (Croy et al., 2013). Thus, we hypothesize that smelling the chemosignal androstenedione via the VNO elicits activation of the hypothalamus. Our findings of reduced olfactory sensitivity in patients with ED support vomeronasal identification of pheromones and possible sexual behavior. In addition, our multi-element regression analyses determined that patients with an impaired sense of smell had a 16.479-fold risk for ED. We believe that the VNO has gradually degraded to non-organ form as a result of human evolution, yet it functions as olfactory cells that detect pheromones controlling human sexual activity.

Taken together, these results support the notion that olfactory function is associated with ED in males. VNO identification of pheromones to regulate sexual behavior will allow us to further explore the hypothesis that improving olfactory sensitivity can improve sexual experience. RDs and ED have obvious superimposing effects on ODs. Patients with RD do not typically report intimate ED problems, so routine medical care providers should inform about this common effect and ask for consultation.

## Data Availability Statement

All datasets generated for this study are included in the article.

## Ethics Statement

This study was approved by the Research Ethics Committee of the Institute of Basic Research in Clinical Medicine, The Third Affiliated Hospital of Sun Yat-sen University.

## Author Contributions

QY, HD, JF, and YZ conceived and designed the study. JF, HD, WK, WZ, GM, TH, SL, and YX acquired the data. JF and HD analyzed and interpreted the data, and drafted the manuscript. QY and YZ critically revised the manuscript for important intellectual content. All authors approved the final manuscript.

## Conflict of Interest

The authors declare that the research was conducted in the absence of any commercial or financial relationships that could be construed as a potential conflict of interest.
